# Case of Nasal Sarcoma, with Remarks

**Published:** 1902-01

**Authors:** Dunbar Roy

**Affiliations:** Clinical Professor of Eye, Ear, Nose and Throat Diseases in the Atlanta College of Physicians and Surgeons; Oculist and Aurist to the Grady Hospital; Fellow of the American Laryngological, Rhinological and Otological Society; Associate Member of the American Ophthalmological Society, etc., Atlanta, Ga.


### CASE OF NASAL SARCOMA, WITH REMARKS.

By DUNBAR ROY, A.B., M.D.

Clinical Professor of Eye, Ear, Nose and Throat Diseases in the Atlanta College of Physicians and Surgeons; Oculist and Aurist to the Grady Hospital; Fellow of the American Laryngological, Rhinological and Otological Society; Associate Member of the American Ophthalmological Society, etc., Atlanta, Ga.

The sarcomata within the last few years seem to be on the increase. This may be due to the fact of a closer microscopic examination being made than formerly of all suspected tumors, especially those occurring in the nasal passages. Nor can rhinologists be too diligent in this direction. It is not my intention to go into the pathology of the sarcoma, but simply to add a few personal experiences to the report of a case.

Warren, in his late book on Surgical Pathology, states that "sarcoma of the nasal passages is not a very rare disease." Bosworth has collected forty-one published cases, but I believe that this number is but a small proportion of the cases which have occurred. If statistics were really obtainable they would show a much larger percentage of these growths than is at present the case.

The round cell and alveolar forms of sarcoma seem to be the prevailing types of the growth occurring in the nasal passages, although there are sometimes seen the fibrosarcoma, myxosarcoma and even angio and melanosarcoma. Some writers hold that it is extremely rare for a sarcoma to originate primarily in the nasal passages, but that such nearly always has its origin in one of the contiguous cavities, especially the maxillary antrum. This is a difficult question to decide, since the rhinologist so rarely sees a case and follows it from its very incipiency.

Warren says that the tumors are pediculated and occur with about equal frequency on the outer and inner wall of the nasal passages. Histories of reported cases do not seem to substantiate this statement, rather showing a greater frequency on and around the turbinates than at any other point.

The average age at which the disease appears is about forty years, and is seen about equally between males and females.

Warren further says that sarcoma does not appear to show the same malignant tendencies in the nasal passages that it does in other localities. There have been several cases reported where the disease had not shown a recurrence several months after its removal.

The etiology of sarcoma appears as yet to be enveloped in obscurity, but the majority of pathologists incline to the theory of Conheim that it depends upon a disturbance of embryonic tissue.

Nasse says that trauma is more frequently the cause of sarcoma than of any other tumor, and observation teaches that there is certainly a close relationship between the two, especially in this class of tumors occurring in the nose. In my own mind I firmly believe that there is a possibility of benign growths in the nasal passages, such as the myxomata, being transformed into malignancy through traumatic irritation. Such a statement, however, is difficult to verify, since I have been unable to find any authentic case which has been traced from its very incipiency to the point where it has become malignant. There is enough evidence to show that this possible transformation has been seriously considered by others. Last year Wurdemann, of Milwaukee, reported a case of sarcoma of the nasal passages in which he seemed very much inclined to the belief that it was a transformation from benign to malignant neoplasm through rough instrumental manipulation.

If such then be a possibility, how necessary then is it for the rhinologist to be certain of the character of every growth which he finds in the nose and the radical removal of the same in the very beginning when there are the least signs of malignancy and this, too, with as least traumatism possible.

The prognosis in these cases is anything but favorable, and any treatment which will stay the progress of the growth should be welcomed.

The case which I will now report is done so for two reasons: first, because of the probable transformation of a benign into a malignant growth, and, secondly, because of the surgical method used in the treatment.

Miss B., age twenty-eight, from the southern part of the State, was referred to me in June, 1899. She gave the following history: Family history was most excellent. The patient herself had always been perfectly healthy up to February, 1899, when the right nasal cavity began to stop up and cause difficulty in breathing. She consulted her family physician, who told her that she had a polypus and at the same time removed it with a pair of scissors. In a short time the growth returned and he again removed it. After a short interval, the nasal cavity still being stopped, she went to a neighboring city to consult a surgeon. He told her it was a surface polypus and could be removed. She remained there a month and at various times the growth was curetted and torn away. There was always excessive hemorrhage at each operation. At the end of a month the patient returned home with the assurance that the place would soon heal up. However, the growth gradually increased in size, the whole side of the face on that side swelling enormously, and a swelling also appeared in the roof of the mouth.

On June 6, four months after the appearance of the first symptoms, she came to Atlanta. On examination I found the right nasal cavity septum and right antrum involved with the growth which was evidently a sarcoma. The mother was advised that the case was hopeless, but at her solicitation I began a trial of Coley's mixture of erysipelatous toxines obtained from Parke, Davis & Co. This was used for two weeks with decided reactions but no change in the growth. At this time Dr. W. P. Nicolson, a general surgeon, saw the case with me and advised as a last resort, not as curative but as palliative, the cutting off of the blood supply to the tumor by ligating both external carotids. The consent was given and on June 19, at St. Joseph's Infirmary, Dr. Nicolson ligated both external carotids, the operation being accomplished in half an hour. The patient reacted nicely from the operation. On the second day the temperature rose to 102 F., but only for a day. The only thing complained of was pain on deglutition on account of the soreness in the pharyngeal muscles. The growth seemed to decrease some in size after the operation and began to lose its fiery red color so noticeable previously. The color became more of a purplish hue. For a few days there seemed to be an improvement and the patient complained much less of the throbbing sensation. No material change being noticed at the end of two weeks and the patient gradually growing weaker every day, she was taken home and died on August 16, seven months after the appearance of the first symptoms.

The prognosis in this case was unfavorable from the very beginning. It is certainly one which adds something to the idea of transformation of benign into malignant neoplasms. The question as to whether the growth, considered in the beginning as a mucous polypus, was not even then malignant, could not be answered, as no microscopic examination was made, but it at least opens up the question for further investigation.

Another interesting point was the rapidity of its growth within the space of six months. Kyle, in his late text-book, says that nasal sarcoma are of slow growth, and this view is held by the majority of writers.

The symptom of severe bleeding, whenever any growth within the nasal cavities is touched, should make us very suspicious, and such should be removed early and in the most radical way possible.

I am opposed to the vigorous curetting of any growth in the nose, for if transformation of benign into malignant growths is possible, then curetting opens up the very best chance for such to occur.

The thorough cutting off of the blood supply to these growths by ligating the vessels supplying the same has recently been strongly advocated. One case, of course, is not sufficient to draw deductions as to the curative effect of any treatment. In the case reported both external carotids were tied so as to remove all possible source of bilateral anastimosis. Our operation produced no lasting beneficial effect. Such an operation might be effective in the early stages of the growth, but I do not think it will be of any benefit after marked infiltration of the tissues has occurred.

The use of the toxins was not persisted in because, as is well known, such are effective by destroying the tumor, and if such had happened in this case, the disfigurement would have been worse than death.

*Grand Opera House.*

